# First Clinical Report on Comparative Treatment and Survival Outcomes in Second Cancers after Primary Head and Neck Cancer: A Population-Based Study

**DOI:** 10.7759/cureus.1284

**Published:** 2017-05-29

**Authors:** Xin Wang, Elizabeth A Mauer, Paul Christos, Julia Manzerova, A. Gabriella Wernicke, Bhupesh Parashar

**Affiliations:** 1 Stich Radiation Oncology, NewYork-Presbyterian/Weill Cornell Medical Center; 2 Division of Biostatistics and Epidemiology, Department of Healthcare Policy and Research, New York-Presbyterian/Weill Cornell Medical Center

**Keywords:** seer, head and neck cancer, second primary malignancy, treatment types, survival

## Abstract

**Introduction:**

To compare patients’ survival of second primary malignancy (SPM) after head and neck squamous cell carcinoma (HNSCC).

**Methods:**

The Surveillance, Epidemiology, and End Results (SEER) database was utilized (1973-2011). The Kaplan-Meier method with log-rank test was used to compare the overall survival (OS) and cause-specific survival (CSS) among treatment methods from the time of diagnosis of SPMs. Cox proportional regression models were used to adjust the impact for risk factors on CSS.

**Results:**

A total of 3,038 patients were identified (5-yr OS 22.6% (21.0-24.3%)). For head and neck (HN) SPMs, the patients who received ‘conservative surgery with radiation’ had the best 5-yr OS (65.2% (48.9-86.9%)); and the ‘conservative surgery’ group had the best 5-yr CSS (89.9% (85.6-94.5%)). For lung SPMs, the ‘radical surgery’ group showed the best survival (2-yr OS 60.8% (56.0-66.1%), 2-yr CSS 70.6% (65.8-75.8%), respectively). Esophagus SPMs had poor prognosis, with no difference among the treatment groups. In lung SPMs, younger age (p<0.001) and black race (p<0.05) were most favorable CSS predictors.

**Conclusions:**

The prognosis of SPMs after HNSCC is worse compared with corresponding primary tumor. Conservative surgery with or without radiation showed the most favorable outcomes in HN SPMs.​

## Introduction

Head and neck (HN) cancer patients are known to be at significantly elevated risk of second primary malignancies (SPMs), compared with the matched population and SPMs are the leading cause of mortality (30% [1-2]) among long-term head and neck squamous cell carcinoma (HNSCC) survivors. Effective and successful SPM treatment is therefore a crucial part of long-term HNSCC management.

The concept of ‘field cancerization’, which was first introduced by Slaughter, et al. [3] in 1953, has been used to explain the high occurrence of SPMs in HNSCC. The classic view of ‘field cancerization’ is that exposure of environmental carcinogens, such as tobacco and alcohol use, may induce premalignant disease in large mucosal area and elevate the secondary cancer risk throughout the aero-digestive tract. Additional studies have further confirmed that some SPMs share a similar genetic pattern with the index tumor [4]. Other risk factors, such as aging and human papillomavirus (HPV)-seronegative status, etc. have been proven to significantly increase the incidence of SPMs [5-6]. The most common site of HNSCC SPMs is lung, followed by HN, and esophagus [5, 7-8], attributing to about 60% of all SPMs combined.

Patients who develop an SPM tend to have a significantly worse survival, compared to those who do not [7]. Head and neck SPMs have a relatively better prognosis than SPMs arising in lung or esophagus, with a 5-yr survival of 61%, 19%, and 0%, respectively [2, 9]. Lung SPM patients after an HNSCC primary tumor have a worse prognosis compared with the matched general population with primary lung cancer, regardless of the histology [10]. All these findings highlight the importance of better understanding and management of HNSCC SPMs.

In this population-based study, we aim to compare the survival outcomes across treatment types in HNSCC patients who developed an HN, lung, or esophagus SPM and to determine whether there is any association between risk factors and the survival in SPM patients.

## Materials and methods

### The Surveillance, Epidemiology, and End Results program and Multiple Primary–Standardized Incidence Ratios session

The Surveillance, Epidemiology, and End Results (SEER) program of the National Cancer Institute (NCI) is an authoritative source of information on cancer incidence and survival in the US, which routinely collects data on patient demographics, tumor site, morphology, stage at diagnosis, course of treatment, and follow-up for vital status, with quality control routinely performed since 1973 and now covers approximately 28% of the US population [11]. The NCI does not require institutional board approval for the use of SEER data.

The Standardized Incidence Ratios’ (MP-SIR) session is a session under the SEER program, in which a defined cohort of people previously diagnosed with cancer is followed through time to compare their subsequent primary cancer experience to the matching general population [12]. By extracting the detailed information of the secondary cancers, one can also look into the risk factors, management techniques, and survival outcomes of specific SPMs [13].

### Study population and treatment categories

The SEER*Stat 8.2.1 software package (SEER, National Cancer Institute, MD, USA) and the MP-SIR session were used to identify patients who were diagnosed with a primary HNSCC and then developed SPMs between 1973 and 2011 in the nine registries of the SEER program. Patients with HNSCC were defined using the International Classification of Diseases for Oncology, third edition (ICD-O-3), histology codes for squamous cell carcinoma (8070-8078), and subsite codes for head and neck cancer (oral cavity, oropharynx, hypopharynx, larynx, and nasopharynx). All patients included were in 'active follow-up' or 'originally inactive, then active follow-up' as defined in the database. Given the relatively small study population for each SPM subsite, the stage at presentation was categorized according to the SEER historic stage A codes as ‘localized’ (localized without lymph node involvement or distant metastases, N0M0), ‘regional’ (locally advanced or lymph node-positive without distant metastases, N+M0), or ‘distant’ (distant metastases, any N M1) [14]. SPM was defined as an invasive solid tumor developing ≥2 months after an index HNSCC as per National Cancer Institute criteria [15]. Patients with HN, lung, and esophagus SPMs were drawn from the case listing sheet, and the ones who died from the index tumor, developed a third malignancy, or had an un-staged disease were excluded.

Two SEER variables ‘Site specific surgery (1983-1997)’ and ‘Surgery of the primary site (1988+)’ were recoded together to define and categorize the surgical treatment received. Radiation therapy (RT) was limited to ‘Beam radiation’ (SEER code 1) and ‘Combination of beam and implants or isotopes’ (SEER code 4). For HN SPMs, treatment categories were defined as: ‘conservative surgery (CS, SEER codes 10-30, including electroautery, cryosurgery, laser surgery, local excision, partial pharyngectomy, tonsillectomy, and partial laryngectomy, etc., but excluded excisional biopsy)’, ‘radical surgery (RS, SEER codes 40-90, including radical excision with/without lymph nodes dissection, total laryngectomy, and pharynolaryngectomy, etc.)’, ‘radiation (RT)’, ‘conservative surgery with radiation (CSRT)’, ‘radical surgery with radiation (RSRT)’, and ‘no treatment’ group. We acknowledge that for pharynx, in ‘Surgery of the primary site (1998+)’ manual, code 30 also includes total pharyngectomy. However, for simplicity, we categorized it in the CS group. CS and CSRT groups (including local tumor destruction as laser ablation, cryosurgery, electroautery, and photodynamic therapy with/without radiation) were excluded from lung and esophagus SPMs because of atypical treatment patterns and very small patient number. For lung SPMs, RS was defined as SEER codes 20-90, included wedge resection, segmental resection, lobectomy, pneumonectomy, radical pneumonectomy, and extended pneumonectomy. For esophagus SPMs, RS was defined as SEER codes 40-90 in the ‘Site specific surgery (1983-1997)’ manual and 30-90 in the ‘Surgery of the primary site (1998+)’ manual and included partial/total esophagectomy with/without laryngectomy and/or gastrectomy.

### Statistical analysis

Descriptive statistics were calculated to characterize the study cohort in relation to demographic factors, social-economic status, and tumor characteristics. The primary endpoint was cancer-specific survival (CSS). For CSS, the patients with deaths due to causes other than cancer were censored. A secondary endpoint was overall survival (OS). Time for both CSS and OS was calculated as time in months from the diagnosis of the SPM tumor to the date of death from any cause, or to the date of last follow-up if a patient did not have a recorded death. Kaplan-Meier survival analyses were performed to evaluate CSS and OS by SPM tumor staging and treatment group, stratified by SPM subsite. All analyses were used to produce relevant survival estimates and to employ the log-rank test for comparison of CSS/OS between the different groups of interest.

Unadjusted Cox proportional hazards regression analyses were performed to evaluate the impact of all demographic, social-economic, and tumor factors on CSS, stratified by SPM subsite. Multivariable Cox proportional hazards regression analyses was then performed with all variables included that were significant at the 0.20 alpha level from the unadjusted analyses. ‘Age at index tumor’ and ‘year of index tumor’ were excluded because of their collinearity with corresponding SPM data. Competing-risks survival regression was also performed to check the multivariable CSS hazard ratios by accounting for the competing event of death due to causes other than cancer (based on Fine and Gray’s proportional subhazards model). All p-values are two-sided with statistical significance evaluated at the 0.05 alpha level. Ninety-five percent confidence intervals (CI) for unadjusted and adjusted HRs and subhazard ratios were calculated to assess the precision of the obtained estimates. All analyses were performed using R-3.2.0 for Windows 64 bit (R-statistics.com, Vienna, Austria).

## Results

### Description of study population

A total of 3,138 patients with a median follow-up of 12 months were identified, of which, 1,056 patients developed an HN SPM (33.7%), 1,717 patients developed a lung SPM (54.7%), and 265 patients developed an esophagus SPM (8.4%). The patient and tumor characteristics are summarized in Table 1. The median age for index and secondary tumor was 61 years, and 69 years, respectively. The median time interval from the diagnosis of an index tumor to an SPM was 70 months (range 2-242 months). It was found that 51.5% had a localized or regional (42.7%) index tumor. In patients with lung SPMs, 45.1% of patients (N=775) had distant disease at diagnosis (Table 1).

**Table 1:** Descriptive Characteristics by SPM Subsite (1973-2011) Abbreviations: CS, conservative surgery; CSRT, conservative surgery with radiation; HN, head and neck; mo, months; RS, radical surgery; RSRT, radical surgery with radiation; RT, radiation therapy; SPM, second primary cancer; yo, years.

	Patients by Subsite						
	All Sites		HN		Lung	Esophagus	
	N (%)		N (%)		N (%)	N (%)	
Characteristic	N=3038		N=1056		N=1717	N=265	
Median Follow-up Time (mo)	12		30		8	8	
Gender							
Male	2221 (73.1)		763 (72.3)		1244 (72.5)	214 (80.8)	
Female	817 (26.9)		293 (27.8)		473 (27.5)	51 (19.3)	
Ethnicity							
White	2625 (86.4)		943 (89.3)		1497 (87.2)	185 (69.8)	
Black	289 (9.5)		75 (7.1)		155 (9.0)	59 (22.3)	
Other	124 (4.1)		38 (3.6)		65 (3.8)	21 (7.9)	
Age of Index Tumor (yo)							
≤45	216 (7.1)		119 (11.3)		82 (4.8)	15 (5.7)	
46-55	711 (23.4)		258 (24.4)		377 (22.0)	76 (28.7)	
56-65	1161 (38.2)		354 (33.5)		704 (41.0)	103 (38.9)	
66-75	743 (24.5)		241 (22.8)		443 (25.8)	59 (22.3)	
＞75	207 (6.8)		84 (8.0)		111 (6.5)	12 (4.5)	
Age of SPM (yo)							
≤45	54 (1.8)		37 (3.5)		12 (0.7)	5 (1.9)	
46-55	275 (9.1)		119 (11.3)		125 (7.3)	31 (11.7)	
56-65	866 (28.5)		317 (30.0)		465 (27.1)	84 (31.7)	
66-75	1125 (37.0)		327 (31.0)		699 (40.7)	99 (37.4)	
＞75	718 (23.6)		256 (24.2)		416 (24.2)	46 (17.4)	
Year at diagnosis of Index Tumor							
1973-1980	408 (13.4)		186 (17.6)		170 (9.9)	52 (19.6)	
1981-1990	1015 (33.4)		319 (30.2)		580 (33.8)	116 (43.8)	
1991-2000	1042 (34.3)		333 (31.5)		648 (37.7)	61 (23.0)	
2001-2011	573 (18.9)		218 (20.6)		319 (18.6)	36 (13.6)	
Year at diagnosis of SPM							
1973-1980	56 (1.8)		45 (4.3)		N/A	11 (4.2)	
1981-1990	474 (15.6)		208 (19.7)		197 (11.5)	69 (26.0)	
1991-2000	1126 (37.1)		296 (28.0)		723 (42.1)	107 (40.4)	
2001-2011	1382 (45.5)		507 (48.0)		797 (46.4)	78 (29.4)	
Median Months since Index Tumor (mo)	70.00		73.00		68.00	67.00	
Marital Status							
Unmarried	1176 (38.7)		372 (35.2)		698 (40.7)	106 (40.0)	
Married	1730 (56.9)		606 (57.4)		976 (56.8)	148 (55.8)	
Unknown	132 (4.3)		78 (7.4)		43 (2.5)	11 (4.2)	
% At Least Bachelors Degree (2000)							
≤25		1187 (39.1)		397 (37.6)	701 (40.8)		89 (33.6)
＞25		1851 (60.9)		659 (62.4)	1016 (59.2)		176 (66.4)
Median Family Income (in Ks, 2000)							
≤50		949 (31.2)		329 (31.2)	542 (31.6)		78 (29.4)
＞50		2089 (68.8)		727 (68.8)	1175 (68.4)		187 (70.6)
% Families below Poverty (2000)							
>=10		599 (19.7)		195 (18.5)	328 (19.1)		76 (28.7)
<10		2439 (80.3)		861 (81.5)	1389 (80.9)		189 (71.3)
Index Tumor Staging							
Localized		1564 (51.5)		645 (61.1)	813 (47.4)		106 (40.0)
Regional		1297 (42.7)		359 (34.0)	795 (46.3)		143 (54.0)
Distant		177 (5.8)		52 (4.9)	109 (6.3)		16 (6.0)
SPM Tumor Staging							
Localized		1136 (37.4)		619 (58.6)	420 (24.5)		97 (36.6)
Regional		981 (32.3)		366 (34.7)	522 (30.4)		93 (35.1)
Distant		921 (30.3)		71 (6.7)	775 (45.1)		75 (28.3)
Treatment							
CS		270 (8.9)		270 (25.6)	N/A		N/A
RS		741 (24.4)		307 (29.1)	384 (22.4)		50 (18.9)
RT		909 (29.9)		163 (15.4)	623 (36.3)		123 (46.4)
CSRT		45 (1.5)		45 (4.3)	N/A		N/A
RSRT		204 (6.7)		83 (7.9)	85 (5.0)		36 (13.6)
None		869 (28.6)		188 (17.8)	625 (36.4)		56 (21.1)
Vital Status							
Alive		630 (20.7)		403 (38.2)	202 (11.8)		25 (9.4)
Noncancer Deaths		685 (22.5)		360 (34.1)	289 (16.8)		36 (13.6)
SPM Specific Deaths		1572 (51.7)		209 (19.8)	1168 (68.0)		195 (73.6)
Unknown Cause Deaths		151 (5.0)		84 (8.0)	58 (3.4)		9 (3.4)

### Overview of SPM survival by subsite

**Figure 1:**
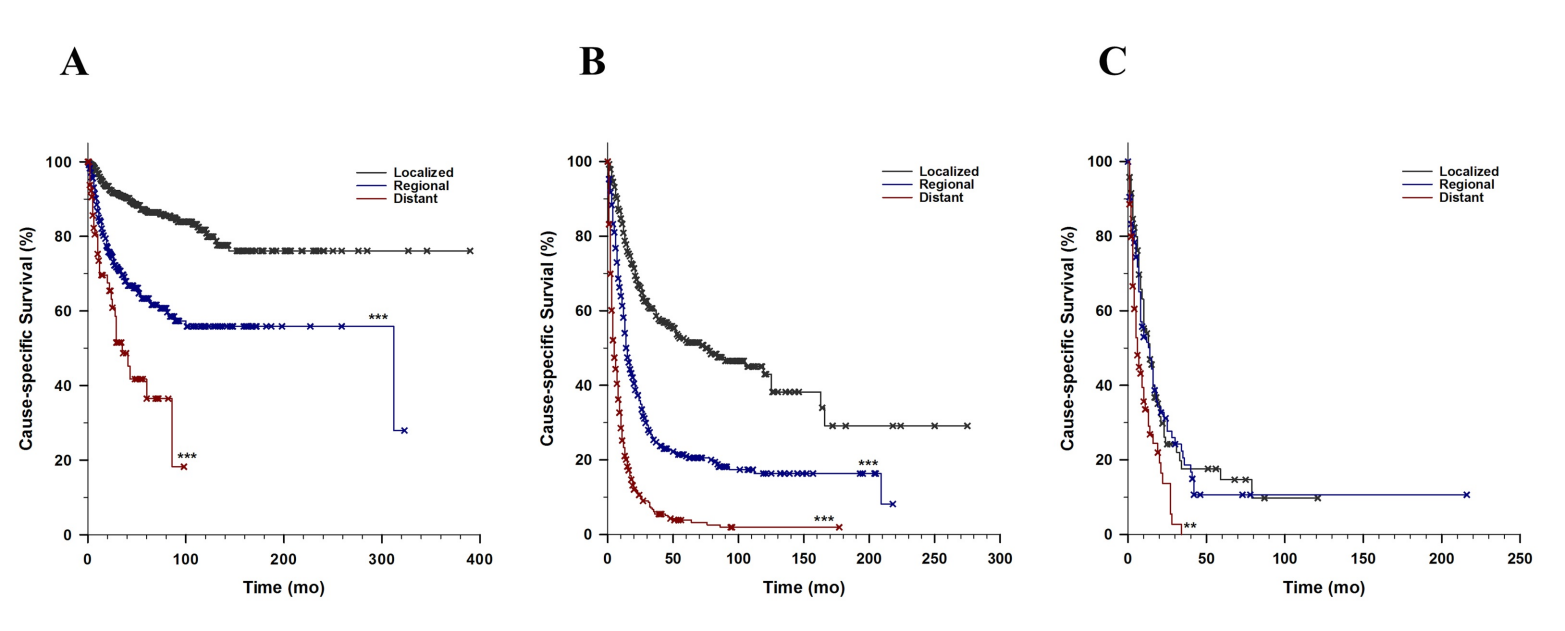
Cause-specific Survival by Stage A. Cause-specific survival of HN SPMs by SPM Staging (Log-rank <0.001) B. Cause-specific survival of lung SPMs by SPM Staging (Log-rank <0.001) C. Cause-specific survival of esophagus SPMs by SPM Staging (Log-rank<0.001) ** p<0.01, *** p<0.001 compared with corresponding localized SPMs

The study cohort had a median OS of 14 months (13-15 months) and a median CSS of 22 months (20-26 months). HN SPMs (Table 2) 5-yr OS was 44.0%, (40.7-47.5%) and 5-yr CSS 75.6%, (72.4-78.9%). Patients with esophagus SPM had a 5-yr OS and 5-yr CSS less than 10.0%. The SPM staging was closely correlated with survival, except esophageal SPMs where there was no survival difference between localized versus non-localized SPMs (Figures 1A-1C).

**Table 2:** Overview of Survival Outcomes by Subsite Abbreviations: CI, confidence interval; CSS; cause-specific survival; HN, head and neck; HR, hazard ratio; OS, overall survival; R, reference. ***p<0.001

Site	5-yr OS	95% CI	5-yr CSS	95% CI	HR (CSS)
	(%)	(%)	(%)	(%)	
All Sites	22.6	21.0-24.3	38.8	36.8-40.9	NA
HN (R)	44.0	40.7-47.5	75.6	72.4-78.9	1.000
Lung	12.4	10.8-14.2	20.5	18.3-23.0	6.152***
Esophagus	4.9	2.7-8.9	8.7	5.1-14.8	7.051***

### Correlation between treatment type and site-specific survival

Head and Neck

For HN SPMs, surgical resection was the most frequently performed treatment (RS 29.1% and CS 25.6%, Table 1). Patients who received CSRT had 2-yr OS of 87.1% (77.1-98.4%) and a 5-yr OS 65.2% (48.9-86.9%), while patients who received CS showed a 5-yr CSS of 89.9% (85.6-94.5%). The RSRT group had 5-yr CSS of 57.9% (46.2-72.6%) (Table 3). Interestingly, the ‘no treatment’ group revealed a comparable survival to RS (5-yr CSS 74.5% (67.6-82.1%)), especially in localized SPMs (unadjusted HR of 0.548 (p<0.1)) (Table 4). 

**Table 3:** Survival of SPMs by Subsite Abbreviations: SPM, second primary cancer; CS, conservative surgery; RS, radical surgery; RT, radiation therapy; CSRT, conservative surgery with radiation; RSRT, radical surgery with radiation; HN, head and neck; R, reference; OS, overall survival; CSS; cause-specific survival; HR, hazard ratio; CI: confidence interval. . p<0.1, * p<0.05, ** p<0.01, ***p<0.001

SPM Site	Treatment	2-yr OS (95% CI)	5-yr OS (95% CI)	2-yr CSS (95% CI)	5-yr CSS (95% CI)	HR
		(%)	(%)	(%)	(%)	CSS
HN	CS	78.3 (73.2-83.8)	57.2 (50.7-64.6)	93.0 (89.6-96.5)	89.9 (85.6-94.5)	0.295***
	RS (R)	61.3 (55.9-67.1)	35.3 (29.9-41.6)	84.5 (80.0-89.2)	72.1 (65.8-78.9)	1.000
	RT	60.0 (52.6-68.4)	35.9 (28.3-45.4)	72.0 (64.8-80.0)	67.4 (59.5-76.3)	1.375 .
	CSRT	87.1 (77.1-98.4)	65.2 (48.9-86.9)	97.5 (92.8-100.0)	82.1 (66.7-100.0)	0.364*
	RSRT	62.4 (52.4-74.2)	36.0 (26.3-49.2)	81.4 (72.6-91.1)	57.9 (46.2-72.6)	1.484 .
	None	63.8 (56.9-71.4)	47.7 (40.4-56.3)	79.9 (73.8-86.5)	74.5 (67.6-82.1)	1.017
Lung	Treatment	1-yr OS (95% CI)	2-yr OS (95% CI)	1-yr CSS (95% CI)	2-yr CSS (95% CI)	HR
		(%)	(%)	(%)	(%)	CSS
	RS (R)	73.2 (68.9-77.9)	60.8 (56.0-66.1)	81.3 (77.3-85.5)	70.6 (65.8-75.8)	1.000
	RT	35.1 (31.5-39.2)	15.0 (12.3-18.2)	40.4 (36.5-44.7)	22.0 (18.5-26.0)	3.765***
	RSRT	75.6 (66.8-85.5)	36.3 (27.1-48.6)	78.9 (70.4-88.3)	39.2 (29.5-52.1)	1.959***
	None	16.4 (13.6-19.7)	7.1 (5.26-9.65)	21,0 (17.8-24.9)	11.6 (8.92-15.1)	6.542***
Esophagus	Treatment	1-yr OS (95% CI)	2-yr OS (95% CI)	1-yr CSS (95% CI)	2-yr CSS (95% CI)	HR
		(%)	(%)	(%)	(%)	CSS
	RS (R)	51.9 (39.7-67.8)	NA	61.4 (48.8-77.3)	NA	1.000
	RT	36.9 (29.1-46.8)	NA	44.0 (35.5-54.5)	NA	1.277
	RSRT	35.5 (22.5-55.9)	NA	47.8 (32.7-69.9)	NA	1.080
	None	26.1 (16.7-40.9)	NA	29.4 (19.1-45.3)	NA	2.019**

**Table 4:** Cause Specific Survival of SPMs by Subsite and Stage Abbreviations: SPM, second primary cancer; CS, conservative surgery; RS, radical surgery; RT, radiation therapy; CSRT, conservative surgery with radiation; RSRT, radical surgery with radiation; HN, head and neck; R, reference; OS, overall survival; CSS; cause-specific survival; HR, hazard ratio; CI: confidence interval. . p<0.1, * p<0.05, ** p<0.01, ***p<0.001

SMP Site		Localized				Regional		
	Treatment	5-yr CCS		95% CI	HR	5-yr CSS	95% CI	HR
		(%)			CSS	(%)		CSS
HN	CS	92.0		87.8-96.4	0.339***	82.8	65.3-100.0	0.304*
	RS (R)	82.7		75.5-90.7	1.000	62.6	52.3-74.8	1.000
	RT	79.7		68.9-92.2	0.982	61.3	50.7-74.1	1.497
	CSRT	80.0		57.4-100.0	0.523	83.0	63.5-100.0	0.346
	RSRT	75.0		50.3-100.0	0.952	60.6	47.0-78.1	1.170
	None	88.0		81.8-94.7	0.548 .	47.4	31.3-71.6	2.671**
Lung	Treatment		2-yr CSS	95% CI	HR	2-yr CSS	95% CI	HR
			(%)		CSS	(%)		CSS
	RS (R)		78.5	72.9-84.4	1.000	62.0	53.7-71.6	1.000
	RT		47.4	37.6-59.7	2.619***	26.0	20.3-33.4	2.625**
	RSRT		75.0	54.1-100.0	1.586	35.6	24.7-51.3	1.500*
	None		36.6	25.3-52.7	4.538***	12.8	7.1-23.1	5.210***
Esophagus	Treatment		2-yr CSS	95% CI	HR	2-yr CSS	95% CI	HR
			(%)		CSS	(%)		CSS
	RS (R)		NA	NA	1.000	NA		1.000
	RT		NA	NA	1.674	NA		0.959
	RSRT		NA	NA	1.407	NA		0.818
	None		NA	NA	2.166 .	NA		1.502

Lung

In the lung SPM cohort, 591 patients (83.5%) were diagnosed with a regional or distant disease and radiation was the most commonly used treatment. It was found that 384 cases (22.4%) (232 localized, 60.4%) received surgical resection as the only treatment (Table 1). The RS group had a 2-yr OS of 60.8% (56.0-66.1) and 2-yr CSS of 70.6% (65.8-75.8%), followed by the RSRT group (Table 3), which remained to be the most superior when categorized into stages (Table 4).

Esophagus

In the esophagus SPM group, (Tables 2-3), 1-yr OS was 51.9%, (39.7-67.8%) and 1-yr CSS was 61.4%, (48.8-77.3%), which was observed in RS group (Table 3).

### Impact on survival

In the multivariable model (Table 5), ‘SPM staging’ was an independent predictor of CSS among all three subsites. ‘Index tumor staging’ was also adversely correlated with survival except for esophagus SPMs. Younger age at diagnosis and being black were correlated with better survival outcome in lung SPM patients.

**Table 5:** Multivariable Analysis of Impact of Factors on CSS by Subsite Abbreviations: SPM, second primary cancer; CS, conservative surgery; RS, radical surgery; RT, radiation therapy; CSRT, conservative surgery with radiation; RSRT, radical surgery with radiation; HN, head and neck; R, reference; CSS; cause-specific survival; HR, hazard ratio; CI: confidence interval. . p<0.1, * p<0.05, ** p<0.01, ***p<0.001

	HN			Lung			Esophagus	
Factors	HR	95% CI		HR	95% CI		HR	95% CI
Age of SPM	1.012	0.999-1.026		1.011**	1.005-1.018			
Months since Index Tumor				1.000	0.999-1.001			
Gender								
Male (referent)	1.000			1.000				
Female	0.760	0.540-1.072		0.926	0.810-1.057			
Ethnicity								
White (R)	1.000			1.000				
Black	1.226	0.736-2.042		0.783*	0.629-0.975			
Other	0.763	0.333-1.749		0.982	0.722-1.337			
Marital Status								
Unmarried (R)	1.000			1.000				
Married	0.827	0.609-1.124		0.914	0.808-1.033		0.767.	0.560-1.031
Unknown	0.964	0.535-1.735		0.966	0.658-1.417		1.005	0.430-2.349
% At Least Bachelors Degree								
≤25 (R)	1.000							
＞25	1.144	0.757-1.730						
Median Family Income (K)								
≤50 (R)	1.000			1.000				
＞50	1.269	0.761-2.118		0.931	0.820-1.057			
% Families below Poverty								
>=10 (R)	1.000							
<10	0.797	0.507-1.254						
Index Tumor Staging								
Localized (R)	1.000			1.000			1.000	
Regional	1.569**	1.168-2.107		1.186**	1.048-1.341		1.122	0.834-1.509
Distant	2.180**	1.252-3.793		1.171	0.904-1.518		0.626	0.325-1.205
SPM Tumor Staging								
Localized (R)	1.000			1.000			1.000	
Regional	2.804***	2.009-3.914		1.985***	1.642-2.400		1.127	0.795-1.598
Distant	5.247***	3.384-8.138		3.427***	2.826-4.156		1.714**	1.182-2.484
Treatment								
CS	0.429**	0.257-0.715						
RS (R)	1.000			1.000			1.000	
RT	1.233	0.842-1.807		2.426***	1.976-2.978		1.200	0.802-1.795
CSRT	0.430	0.156-1.189		NA			NA	
RSRT	1.027	0.656-1.607		1.469*	1.073-2.012		1.013	0.598-1.718
None	1.385	0.924-2.076		3.475***	2.793-4.323		1.624*	1.010-2.612

CS showed the highest CSS after adjusting for all the covariates in all HN SPMs (HR 0.429 compared with RS, p<0.01, Table 5) and by stages (localized SPM HR 0.430, regional SPM HR 0.249 compared with RS, p<0.05, Table 6). Patients with localized HN SPM who received no treatment showed a comparable CSS versus RS (HR 0.685, p>0.1) (Table 6). Surgical intervention was superior compared to all the other groups in lung SPMs. However, in esophagus SPMs, no survival difference was observed among treatment types (Table 5).

**Table 6:** Multivarable Analysis of Impact of Factors on HN SPM CSS by Stage Abbreviations: SPM, second primary cancer; CS, conservative surgery; RS, radical surgery; RT, radiation therapy; CSRT, conservative surgery with radiation; RSRT, radical surgery with radiation; HN, head and neck; R, reference; CSS; cause-specific survival; HR, hazard ratio; CI: confidence interval. . p<0.1, * p<0.05, ** p<0.01, ***p<0.001

	Localized			Regional	
Factors	HR	95% CI		HR	95% CI
Gender					
Male (R)				1.000	
Female				0.609.	0.368-1.008
Ethnicity					
White (R)	1.000				
Black	0.997	0.411-2.422			
Other	2.124	0.504-8.952			
Marital Status					
Unmarried (R)	1.000				
Married	0.545*	0.326-0.911			
Unknown	0.858	0.371-1.983			
% At Least Bachelors Degree					
≤25 (R)				1.000	
＞25				1.608*	1.052-2.457
% Families below Poverty					
>=10 (R)	1.000				
<10	0.787	0.418-1.480			
Index Tumor Staging					
Localized (R)	1.000			1.000	
Regional	1.944*	1.151-3.282		1.618*	1.072-2.442
Distant	3.854***	1.774-8.374		1.395	0.432-4.511
Treatment					
CS	0.430*	0.220-0.838		0.249*	0.075-0.821
RS (R)	1.000			1.000	
RT	1.218	0.593-2.502		1.443	0.861-2.417
CSRT	0.566	0.132-2.423		0.404	0.958-1.704
RSRT	1.112	0.328-3.764		1.068	0.595-1.918
None	0.685	0.342-1.373		2.375**	1.299-4.341

## Discussion

Our population-based study showed outcomes of various treatment modalities for SPMs after primary HN SCC. Based on the results of our study, it seems that aggressive surgical approaches with adjuvant radiation may be the better option compared to more conservative approaches in most patients except esophageal SPMs.

Our study (Supplementary 1) confirmed that the burden of SPMs is elevated in HNSCC patients, with a standardized incidence risk (SIR) of 1.90 (p<0.05) and an absolute excess risk (AER) of 167 per 10,000 person years (PYR) compared to the age-, gender-, and calendar year-matched normal population.

Our study found a 5-yr OS of 22.6% from the time of diagnosis of SPM (Table 2), which is significantly lower than the recent reports on >60% 5-yr OS for HN cancers [16-17]. In addition, SPMs arising in the lung and esophagus had a 5-yr OS of 12.4% and 4.9%, respectively, which is significantly worse than those with an HN SPMs (Table 2) and worse than the reported corresponding primary cancer survival as well [18-19].

For HN SPMs, the management is sometimes complicated by the effects of prior treatment, including anatomical changes, risk of re-irradiation, as well as the multifocal nature of these tumors [20]. Thus, often, they are managed similarly as recurrent diseases. In our study cohort, 66.8% HN SPM patients underwent some sort of surgical resection (Table 1). The unequal survival across conservative and radical surgery groups in HN SPMs was somewhat unexpected, especially in regional SPMs. To our knowledge, there are no studies specifically comparing the outcomes between conservative and radical approaches in HN SPMs. Evidence from observational series in primary and recurrent HN tumors suggests that conservative procedures may be able to offer an alternative option for carefully selected patients [21-22]. For example, Ganly, et al. [22] reported that for early-stage recurrent glottis larynx tumors, partial laryngectomy gives a significantly greater 5-yr survival compared with total laryngectomy. Collectively, our findings raise important questions for surgical treatment selection in HN SPMs after HNSCC.

Interestingly, patients who did not receive any intervention for HN SPMs showed a comparable outcome to surgery (5-yr CSS 88.0%, CSS HR 0.685, p>0.05) (Tables 3-5, Supplementary 2). We are not sure of the reason for this finding except that the retrospective nature of data limits information about histology, chemotherapy or other interventions. A possible explanation may be that ‘no intervention’ preserves the immunological status of the patient to fight cancer progression and such patients may be better if left untreated.

Patients with lung SPMs showed best outcomes when treated with RS alone while patients who received RT only were found to have the worst outcomes (Tables 3-5). This may be due either to advanced stage of presentation or due to conventional RT techniques in the years considered in this data collection. Conventional RT has traditionally been considered inferior to surgical resection in lung cancer treatment outcomes. However, in the last decade, increasing utilization of novel RT techniques such as stereotactic body radiation therapy (SBRT) has resulted in excellent local controls for early stage cancer, and future long term outcome data will likely result in significantly better RT outcomes, compared to conventional RT [23]. Lung SPM patients who had RSRT fared worse compared with those who underwent surgery alone (Tables 3, 5). Some studies in primary lung cancer treatment have shown that postoperative RT may not improve OS [24-25]. A randomized trial from France [26] reported that the non-cancer-related death increased with the RT dose delivered per fraction.

Conventional treatment options for esophagus cancer include surgical resection [27], preoperative concurrent chemoradiotherapy or neoadjuvant chemotherapy followed by resection [28-29]. Our study failed to demonstrate the superiority of any one treatment option over another in the management of esophagus SPMs.

In the diagnostic time-controlled multivariable analysis, ‘SPM staging’ was found to be the survival predictor among all SPMs (Table 5). Blacks did better than whites in lung SPMs (Table 5). However, we acknowledge that race was unequally distributed in our study cohort (155 blacks vs. 1497 whites), which could potentially affect statistical results. Interestingly, consistent with prior studies [30], being married was found favorable in HN and lung SPMs in univariate analysis (Supplementary 3) but not in esophagus SPMs.

As in any SEER-based study, limitations [11, 14] of our study include incomplete information on adjuvant therapy, systemic treatment information, risk factors, coding reliability, and patient migration. However, we hope that the large patient numbers and the information that is available is enough to give us an idea about the management options in this subgroup of patients, and this may be useful for clinical decision making as well as generating a hypothesis for a prospective trial.

## Conclusions

SPMs after an index HNSCC are considerably common and negatively impact the survival. The overall prognosis of SPMs after HNSCC has significantly improved over the years but is still poor compared with corresponding primary tumors. In HN SPMs and lung SPMs, conservative surgery with or without adjuvant radiation may be the best treatment option. Esophageal SPMs showed equivalent outcomes irrespective of the type of treatment. Some SPMs can potentially be left untreated or be managed conservatively.

## Appendices

**Table 7:** Sup 1. Elevated Risk of SPM after HNSCC by Subsite Abbreviations: CI: confidence interval; HNSCC, head and neck squamous cell carcinoma; PYR, persons year at risk; SIR, standardized incidence ratio; SPM, second primary cancer. *P<0.05

	Observed Cases	Expected Cases	SIR		Excess Risk per
Subsite			Rate	95% CI	10,000 PYR
All Sites	20,081	10556.71	1.90*	1.88-1.93	167.31
Lung and Bronchus	5,972	1716.62	3.48*	3.39-3.57	74.75
Head and Neck	4,233	403.55	10.49*	10.18-10.81	67.27
Oral Cavity and Pharynx	3,714	276.47	13.43*	13.00-13.87	60.39
Larynx	519	127.08	4.08*	3.74-4.45	6.88
Esophagus	972	135.58	7.17*	6.73-7.63	14.69

**Table 8:** Sup 2. Survival Between Treated and Untreated HN SPMs Abbreviations: CI: confidence interval; CSS; cause-specific survival; HN, head and neck; HR, hazard ratio; OS, overall survival; R, reference; SPM, second primary cancer.

	All Stages				Localized		
Treatment	5-yr OS (95% CI)	5-yr CCS (95% CI)	HR		5-yr OS (95% CI)	5-yr CCS (95% CI)	HR
	(%)	(%)	CSS		(%)	(%)	CSS
Untreated	47.7 (40.4-56.3)	74.5 (67.6-82.1)	1.217		59.5 (50.9-69.6)	88.0 (81.8-94.7)	0.826
Treated	43.2 (39.6-47.1)	75.8 (72.3-79.4)	1.000		51.3 (46.5-56.6)	86.4 (82.7-90.4)	1.000

**Table 9:** Sup 3. Univariate Analysis of Impact of Factors on CSS by Subsite Abbreviations: CI: confidence interval; CS, conservative surgery; CSS; cause-specific survival; CSRT, conservative surgery with radiation; HN, head and neck; HR, hazard ratio; OS, overall survival; R, reference; RS, radical surgery; RSRT, radical surgery with radiation; RT, radiation therapy; SPM, second primary cancer. . p<0.1, * p<0.05, ** p<0.01, ***p<0.001

	HN			Lung		Esophagus
Factors	HR (95% CI)			HR (95% CI)		HR (95% CI)
Age of Index Tumor						
≤45 (referent)	1.000			1.000		1.000
46-55	1.609 (0.883-2.931)			1.095		0.805 (0.419-1.546)
56-65	2.814*** (1.607-4.930)			1.145		0.921 (0.490-1.732)
66-75	1.952* (1.069-3.564)			1.288 .		0.769 (0.397-1.492)
＞75	1.098 (0.475-2.538)			1.498*		1.241 (0.526-2.925)
Age of SPM						
≤45 (referent)	1.000			1.000		1.000
46-55	2.420 (0.716-8.178)			2.133 . (0.932-4.881)		0.515 (0.195-1.360)
56-65	3.055 . (0.959-9.732)			2.135 . (0.952-4.791)		0.684 (0.274-1.706)
66-75	4.676** (1.477-14.811)			2.219 . (0.991-4.967)		0.648 (0.261-1.606)
＞75	2.777 . (0.858-8.992)			2.890* (1.286-6.495)		0.836 (0.327-2.132)
Months since Index Tumor (Categorical)						
2-36 (referent)	1.000			1.000		1.000
37-72	0.936 (0.644-1.361)			1.112 (0.947-1.307)		1.524* (1.043-2.228)
73-108	0.900 (0.604-1.340)			1.042 (0.874-1.242)		1.107 (0.733-1.671)
109-144 (>108 for Esophagus)	0.929 (0.589-1.466)			1.183 . (0.969-1.445)		1.238 (0.844-1.816)
145-180	0.667 (0.352-1.265)			1.121 (0.886-1.420)		
>=181	0.894 (0.546-1.464)			1.198 . (0.990-1.450)		
Gender						
Male (referent)	1.000			1.000		1.000
Female	0.781 (0.564-1.080)			1.092 (0.962-1.240)		1.113 (0.786-1.576)
Ethnicity						
White (referent)	1.000			1.000		1.000
Black	1.632 * (1.048-2.542)			0.838 (0.676-1.038)		1.142 (0.814-1.602)
Other	0.985 (0.437-2.222)			0.956 (0.705-1.298)		1.397 (0.829-2.354)
Year at diagnosis of index tumor						
1973-1980 (referent)	1.000			1.000		1.000
1981-1990	1.020 (0.736-1.412)			0.943 (0.773-1.152)		1.144 (0.799-1.638)
1991-2000	0.465*** (0.316-0.683)			0.913 (0.749-1.113)		1.084 (0.713-1.647)
2001-2011	0.131*** (0.056-0.303)			0.585*** (0.464-0.738)		0.396** (0.214-0.736)
Year at diagnosis of SPM						
1973-1980 (referent) (NA for Lung)		1.000		NA		1.000
1981-1990 (referent for Lung)		1.019 (0.584-1.778)		1.000		0.834 (0.424-1.641)
1991-2000		0.814 (0.470-1.409)		1.041 (0.866-1.252)		1.185 (0.614-2.287)
2001-2011		0.248*** (0.135-0.453)		0.789* (0.654-0.952)		0.550 . (0.273-1.110)
Marital Status						
Unmarried (referent)		1.000		1.000		1.000
Married		0.709* (0.534-0.943)		0.881* (0.783-0.991)		0.808 (0.604-1.081)
Unknown		0.774 (0.439-1.365)		0.958 (0.655-1.403)		0.739 (0.322-1.697)
% At Least Bachelors Degree						
≤25		1.000		1.000		1.000
＞25		1.237 (0.929-1.648)		1.011 (0.900-1.137)		1.074 (0.793-1.454)
Median Family Income						
≤50		1.000		1.000		1.000
＞50		1.249 (0.919-1.696)		0.922 (0.815-1.042)		1.058 (0.771-1.452)
% Families below Poverty						
>=10 (referent)		1.000		1.000		1.000
<10		0.800 (0.577-1.110)		1.025 (0.883-1.191)		1.003 (0.729-1.379)
Index Tumor Staging						
Localized (referent)		1.000		1.000		1.000
Regional		1.733*** (1.304-2.302)		1.096 (0.974-1.234)		1.162 (0.868-1.557)
Distan		2.601*** (1.535-4.405)		0.911 (0.706-1.176)		0.643 (0.341-1.209)
SPM Tumor Staging						
Localized (referent)		1.000		1.000		1.000
Regional		3.219*** (2.378-4.358)		2.262*** (1.885-2.715)		1.146 (0.815-1.610)
Distant		7.171*** (4.738-10.855)		5.869*** (4.925-6.993)		1.944*** (1.361-2.777)
Treatment						
CS		0.295 *** (0.179-0.486)		NA		NA
RS (R)		1.000		1.000		1.000
RT		1.375 . (0.949-1.992)		3.765 *** (3.112-4.555)		1.277 (0.863-1.891)
CSRT		0.364 * (0.133-0.998)		NA		NA
RSRT		1.484 . (0.957-2.303)		1.959 *** (1.442-2.661)		1.080 (0.645-1.808)
None		1.017 (0.688-1.501)		6.542 *** (5.402-7.922)		2.019 ** (1.290-3.160)
